# In vitro assessment of the biocompatibility of chemically treated silicone materials with human lens epithelial cells

**DOI:** 10.1038/s41598-022-08443-2

**Published:** 2022-03-17

**Authors:** Elizabeth C. Y. Kao, Junghee Seo, David J. McCanna, Lakshman N. Subbaraman, Lyndon W. Jones

**Affiliations:** 1 Centre for Ocular Research and Education (CORE), School of Optometry and Vision Science, University of Waterloo, 200 University Avenue West, Waterloo, ON N2L 3G1 Canada; 2 Centre for Eye and Vision Research (CEVR), 17W Hong Kong Science Park, Hong Kong, China

**Keywords:** Biological techniques, Biotechnology

## Abstract

Cytotoxicity testing is a regulatory requirement for safety testing of new ocular implants. In vitro toxicity tests determine whether toxic chemicals are present on a material surface or leach out of the material matrix. A method of evaluating the cytotoxicity of ocular implants was developed using fluorescent viability dyes. To assess the assay’s sensitivity in detecting toxic substances on biomaterials, zinc diethydithiocarbamate (ZDEC) and benzalkonium chloride (BAK) were deposited on silicone surfaces at different concentrations. Human lens epithelial cells (HLEC) were added to the surface of these treated silicone surfaces and were assessed for viability. The viability of both the adherent and non-adherent cells was determined using confocal microscopy with, annexin V, ethidium homodimer, and calcein. Cell metabolism was also evaluated using resazurin and the release of inflammatory cytokines was quantified using a multiplex Mesoscale Discovery platform. Confocal microscopy was shown to be a sensitive assay for evaluating material toxicity, as significant toxicity (p < 0.05) from ZDEC and BAK-treated surfaces compared to the untreated silicone control was detected. Patterns of cytokine release from cells varied depending on the toxin evaluated and the toxin concentration and did not directly correlate with the reduction in cell metabolic activity measured by alamarBlue.

## Introduction

Cytotoxicity testing is essential for determining the biocompatibility of new ocular implant biomaterials. Cytotoxicity tests can potentially detect toxic materials before testing in animals and thus can be used to minimize animal pain and suffering in the development of ophthalmic medical devices^1,2^. The United States Food and Drug Administration (US FDA) and other regulatory agencies throughout the world require cytotoxicity tests to determine the safety of new medical devices. The US FDA and the European Union, for example, have adopted the International Organization for Standardization (ISO) guide 10993 *Biological evaluation of medical devices*. This international standard requires that new medical devices have to be evaluated by the cytotoxicity methods described in ISO 10993-5 *Tests for *in vitro* cytotoxicity*^3,4^.

Since the time when the ISO tests for cytotoxicity were first established, technology has been developed which has improved the understanding of cellular responses to new biomaterials. The alamarBlue assay, which uses reduced resazurin to measure cytotoxicity, assesses the effect a toxin has on cell metabolic activity^5,6^. Using confocal laser scanning microscopy (CLSM) the viability of the cells of interest can be visualized using fluorescent dyes^7^. Also, the release of inflammatory cytokines can be evaluated after treatment with toxins^8^. Thus, it would be beneficial for the assessment of new materials to consider not just the existing ISO standard tests, but to also consider using other methods to strengthen the understanding of the cellular interactions with the material.

Cytotoxic biomaterials can cause harm to adhered cells or to the tissue surrounding an implant, leading to inflammation and the eventual failure of the implant^9,10^. Alternatively, human lens epithelial cell (HLEC) adhesion to a intraocular lens implant followed by cell growth, and movement of the cells to the central visual axis can ultimately lead to posterior capsular opacification (PCO), which can occur after cataract surgery^11–13^. Cataract surgery has also caused toxic anterior segment syndrome (TASS) a condition that can be caused by chemicals on IOL^14,15^ and the incidence of PCO has been shown to be as high as 22% after long-term implantation^16^. In order to reduce TASS and PCO the effect of an implant on HLEC viability, sensitive in vitro methods are necessary to determine the viability thresholds and to accurately predict which biomaterials will most likely be compatible after implantation.

To mimic potential toxins which may be present or leach from a biomaterial’s surface, zinc diethydithiocarbamate (ZDEC) and benzalkonium chloride (BAK) are appropriate chemicals to use, as their toxicity profiles have been well characterized and established both in vitro and in vivo for ocular tissue^17–20^. ZDEC is an accelerator for vulcanization of latex rubber and a known cytotoxic agent that is commonly used as a standard reference material for cytotoxicity and in vivo implant studies^19,21–24^. BAK is a frequently used preservative in ophthalmic eye drops with known toxicity to the eye^20,25–31^. These two chemicals are routinely used as positive controls for in vitro assays and therefore were chosen as test chemicals to assess the validity of the in vitro assays.

By directly exposing HLEC to these toxic chemicals on the surface of a silicone material we are determining the degree at which a model ocular implant material could impact HLEC viability and release inflammatory cytokines after exposure.

## Results

### Confocal analysis of cell viability

Following the 18-h exposure of the HLEC to the treated surface of the silicone, the cells were prepared for confocal analysis. Non-adhered cells on the silicone surface were removed from the surface by the staining solution. The cells that remained on the silicone surface were imaged separately. In the confocal images, live, dead, and apoptotic cells are represented by the colors green (calcein), red (ethidium homodimer), and yellow (annexin V), respectively.

**Figure 1:**
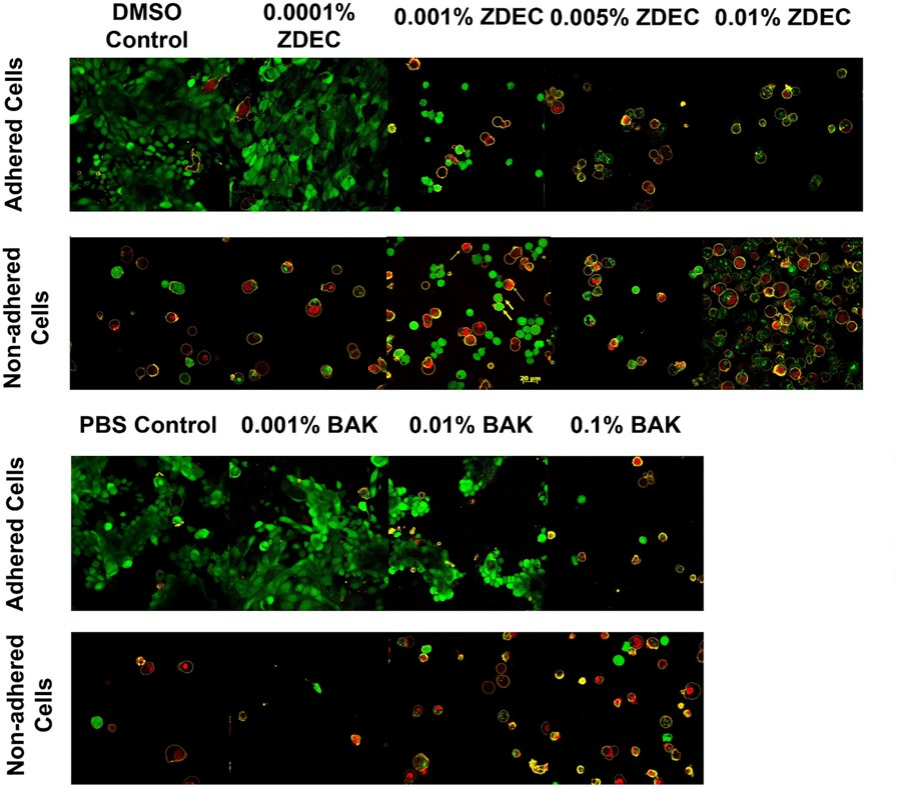
ZDEC and BAK exposed HLEC confocal laser scanning micrographs. Representative confocal laser scanning micrographs showing the effect of the different concentrations of ZDEC and BAK on the viability of the HLEC stained with calcein-AM (green), ethidium homodimer-1 (red), and annexin V (yellow). In addition to the identification of the dye based on their color the dyes’ location also identifies the presence of each dye as well. The calcein dye stains the cytoplasm of the cell and results in typically a uniform distribution throughout the cytoplasm in live cells without compromised membranes. Because ethidium homodimer-1 stains nucleic acid the stain is either centered at the nucleus of the cell or, if the cell is in a later stage of cell death, results in diffuse non-uniform staining of nucleic acid in the cell. Annexin V stains only the cell membrane of the cell. Scale bar is the yellow bar center image.

The confocal images of the HLEC exposed to increasing concentrations of ZDEC and BAK are shown in Fig. 1. The HLEC exposed to concentrations starting at 0.001% ZDEC and 0.1% BAK did not adhere strongly to the treated surface, as the cell morphology changed to a spherical shape, compared to the morphology of an attached cell that is more spindle-like or elongated.

**Figure 2:**
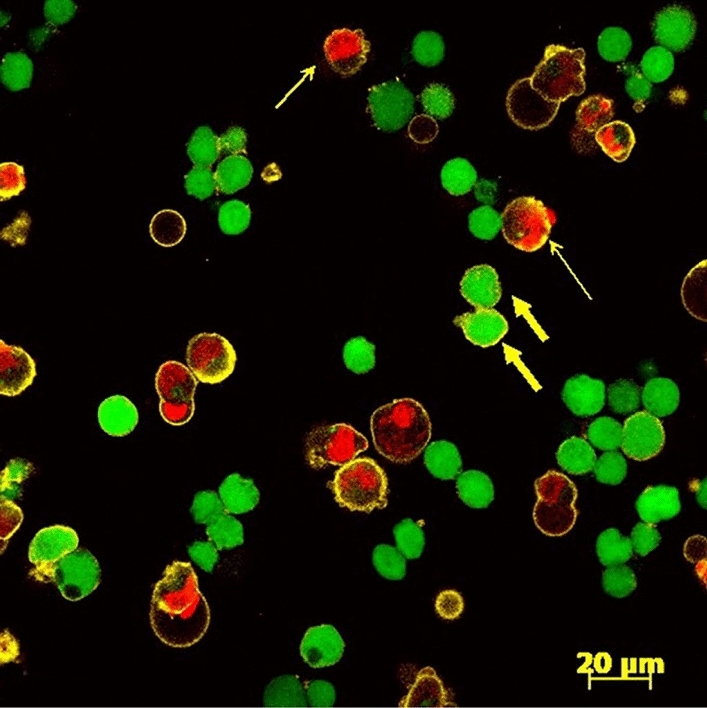
Non-adhered HLEC exposed to 0.001% ZDEC. Representative confocal laser scanning micrograph showing the effect of 0.001% ZDEC on distributions of live (green), dead (red), early apoptotic (green with yellow ring), and late apoptotic (red with yellow ring) cells. A closer look at the apoptotic phenomenon is shown where the wide arrow indicates early apoptosis and narrow arrow indicates late apoptosis.

As shown in Fig. 2, the cells were divided into four categories: live, dead, early apoptotic, and late apoptotic. The live cells are completely green (contain esterases) and have neither red nor yellow; dead cells are red (cell membranes permeable to ethidium) and have no yellow; early apoptotic cells are completely green (contain esterases) with a yellow ring around them (phosphatidylserine translocated to the outer membrane); late apoptotic cells have both red (cell membranes permeable to ethidium) and yellow (phosphatidylserine translocated to outer membrane). Blebbing on the cells also occur on some of the cells which is a common occurrence during apoptosis. In the blebs cytoplasmic material and nuclear material are present on many of the cells. These blebs then form apoptotic bodies. Apoptotic bodies are small vesicles containing cellular components that can be phagocytized by macrophages. Losing contact with neighbouring cells can be another sign of apoptosis. Cells become more separated from each other as the toxin level increases^32^. Color-blind friendly images are shown in Supplementary Figs. 1, 2.

**Figure 3:**
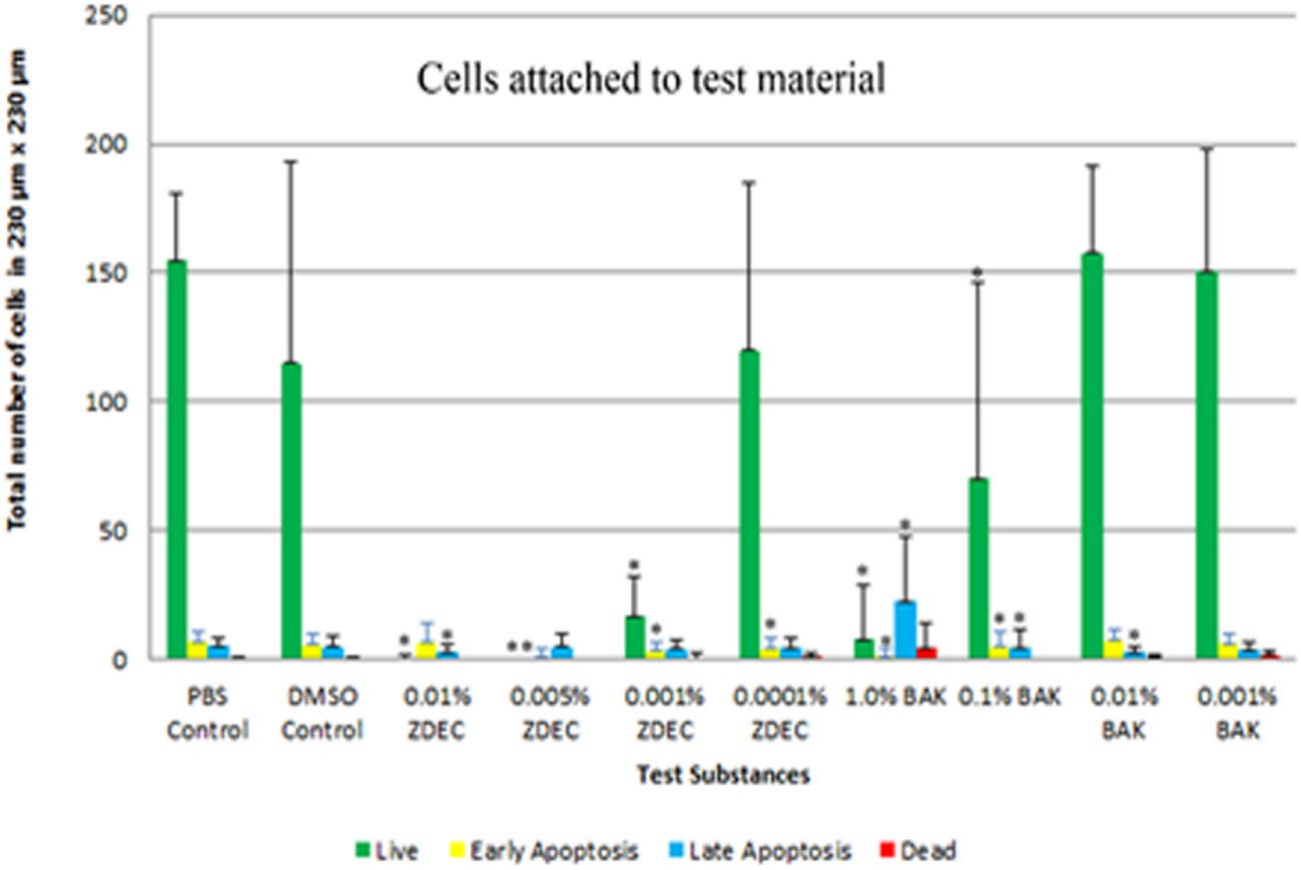
Viability of adhered HLEC to treated silicone surfaces in an area of 230 μm × 230 μm. Viability was quantified by counting the number of live, early and late apoptotic, and dead cells displayed as the first, second, third, and fourth bar of each test substance respectively. n = 15 in each of two separate experiments. *p < 0.05 of the PBS control. Error bars are the standard deviations.

**Figure 4:**
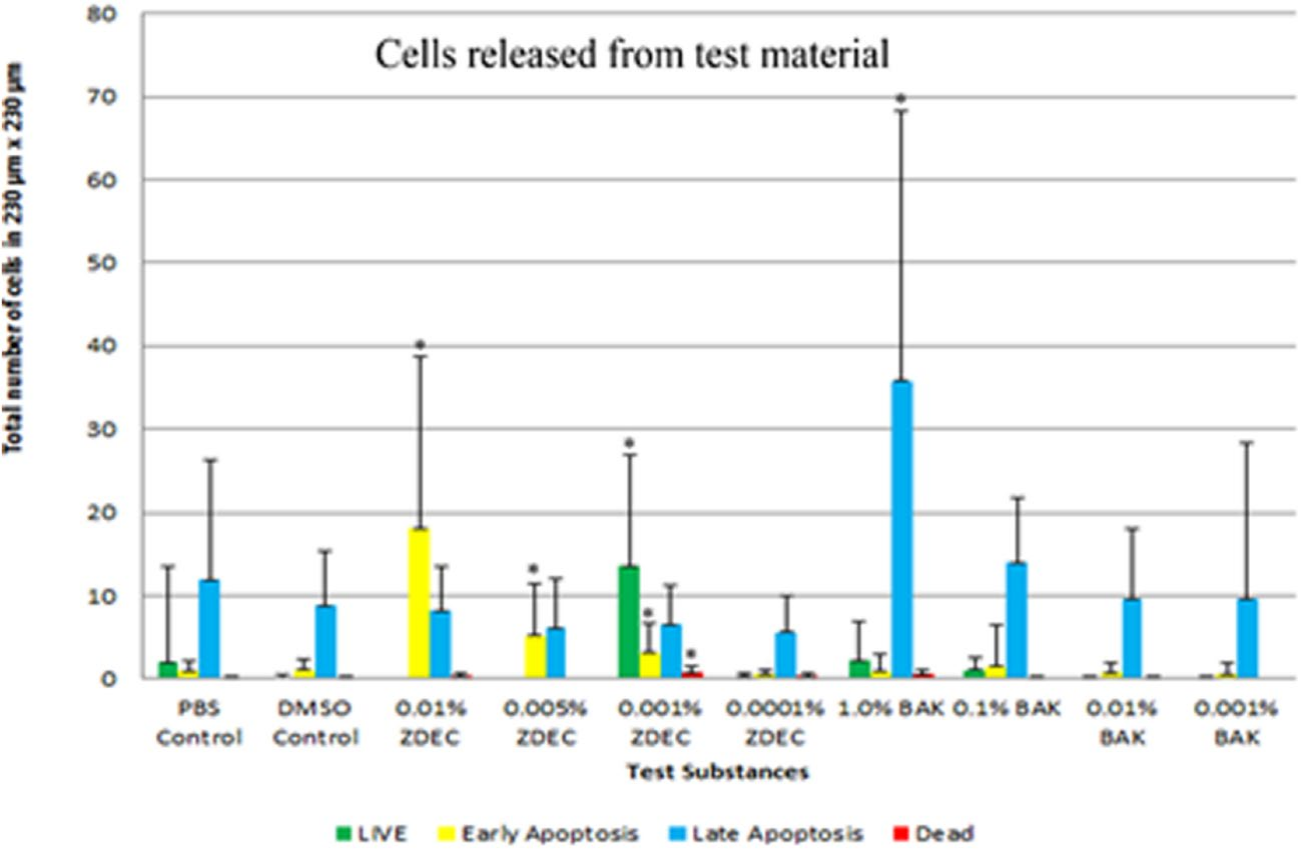
Viability of non-adhered HLEC to treated silicone surfaces in an area of 230 μm × 230 μm. Viability was quantified by counting the number of live, early and late apoptotic, and dead cells displayed as the first, second, third, and fourth bar of each test substance respectively. n = 15 in each of two separate experiments. *p < 0.05 of the PBS control. Error bars are the standard deviations.

Most of the cells in the control group were live and viable, indicated by the greater number of green cells, with a few that were dead or apoptotic due to cell death (Fig. 3). However, the cells which were exposed to the concentrations of ZDEC starting at 0.001% and BAK starting at 0.1% showed a decrease in live cells. Cells that were loosely adherent to the silicone discs that released readily in the sample staining solution were mainly at the later stages of cell death (Fig. 4).

### Release of inflammatory cytokines

**Figure 5:**
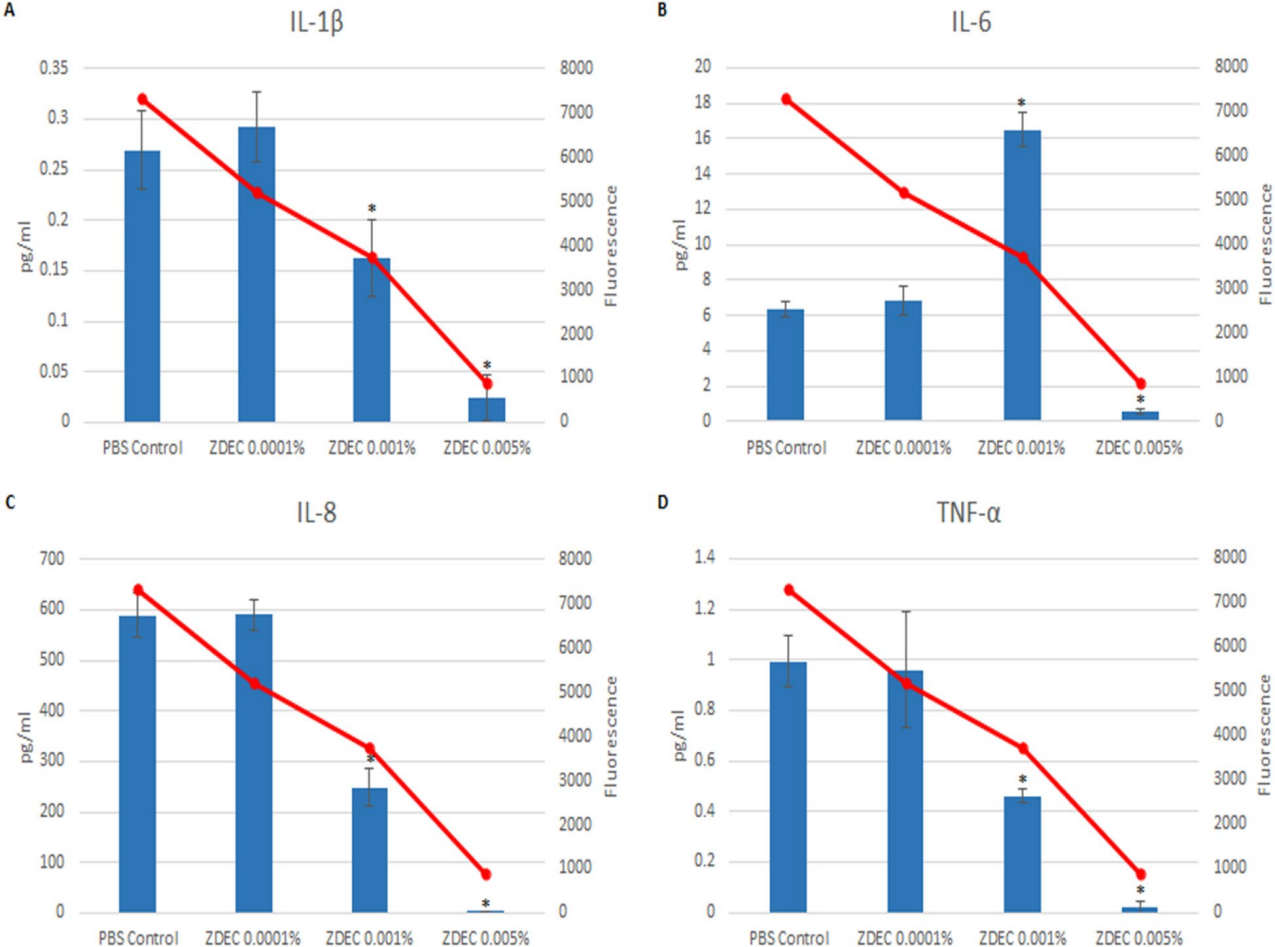
Cytokine release of ZDEC exposed HLEC. Release of four proinflammatory cytokines (bar graphs) and metabolic activity (line graphs) of HLEC exposed to ZDEC treated silicone discs. The four cytokines quantified are IL-1β (**A**), IL-6 (**B**), IL-8 (**C**), and TNF-α (**D**). *p < 0.05 of the PBS control. Error bars are the standard deviations. Experiments performed in triplicate in two separate experiments.

**Figure 6:**
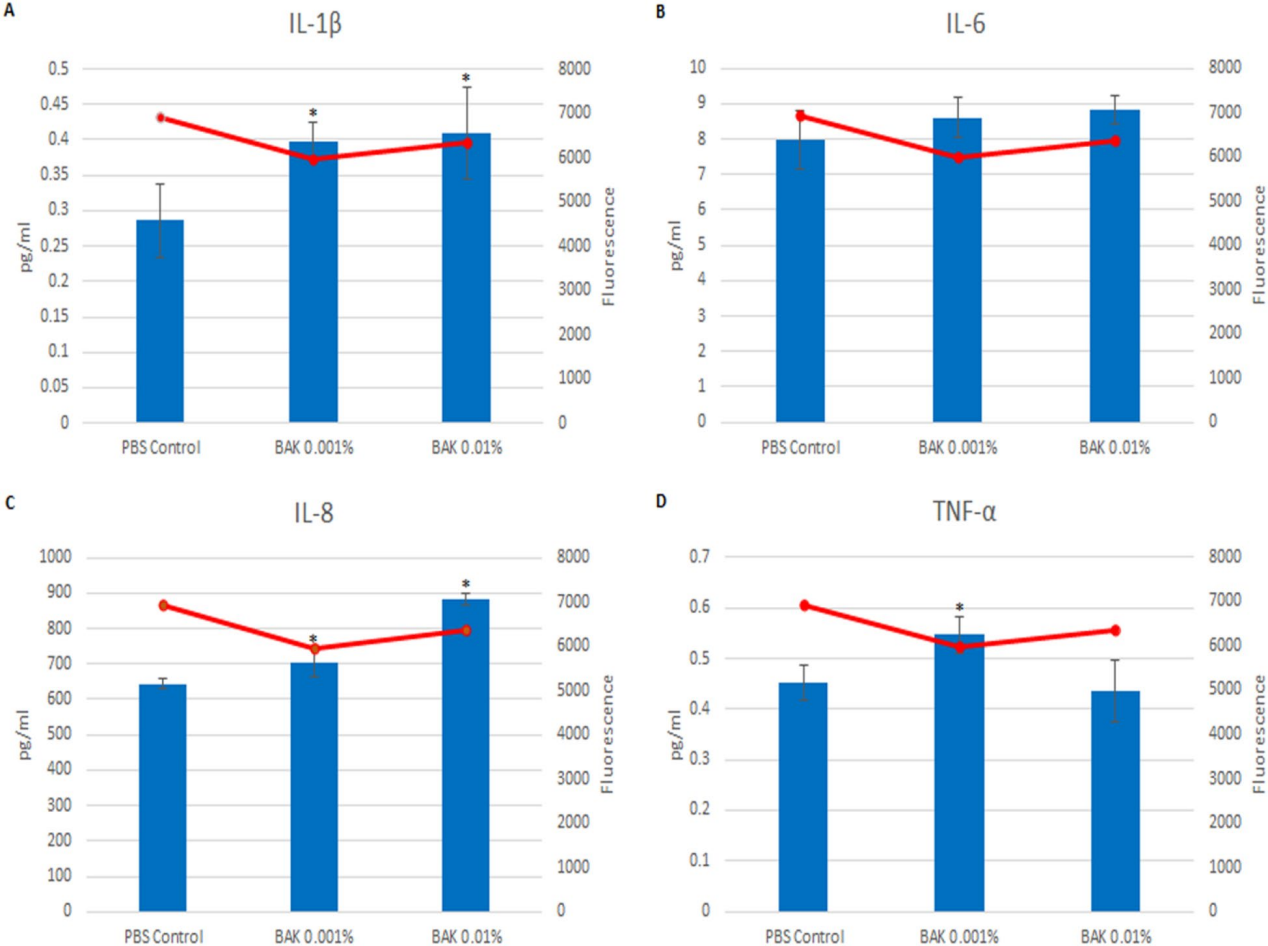
Cytokine release of BAK exposed HLEC. Release of four proinflammatory cytokines (bar graphs) and metabolic activity (line graphs) of HLEC exposed to BAK treated silicone discs. The four cytokines quantified are IL-1β (**A**), IL-6 (**B**), IL-8 (**C**), and TNF-α (**D**). *p < 0.05 of the PBS control. Error bars are the standard deviations. Experiments performed in triplicate in two separate experiments.

Cytokine release from the cells provided a helpful indicator of inflammatory effect. An increase in inflammatory cytokines did not always occur when there was a decrease in metabolic activity. For the HLEC exposed to ZDEC treated discs there was a decrease in the release of the cytokines IL-1β, IL-8 (IL-8 is also known as CXCL8 a chemokine (chemoattractant cytokine), and TNF-α as the ZDEC concentration increased (Fig. 5). However, there was not a linear dose–response with IL-6. The peak of IL-6 release occurred at the intermediate dose (0.001% ZDEC). A different cytokine release profile occurred for BAK (Fig. 6). The pattern of cytokine release showed an increase in the amount of IL-1β and IL-8 in response to an increased concentration of BAK, whereas TNF-α showed an increase at 0.001% and IL-6 showed no increase.

### Metabolic activity of lens epithelial cells

The metabolic activity of the HLEC after 18 h of incubation on the treated discs is shown in Figs. 5 and 6. In Fig. 5, there was a dose-dependent decrease in cellular metabolic activity on the ZDEC treated surfaces. 

## Discussion

The results of this study demonstrated that cytotoxicity methods developed in this evaluation using fluorescent viability dyes could detect the toxicity associated with deposited chemicals on biomaterial surfaces. These in vitro methods could be used in addition to the existing recommended ISO 10993-5 testing procedures to better understand toxicity thresholds and allow for the accurate assessment of a material’s biocompatibility.

The method described in this study can contribute to the understanding and prediction of the viability of cells adhered on an implant, which ultimately points to potential inflammation in vivo*.* According to previous reports, ZDEC was the greatest sensitizer and the most commonly used accelerator in the production of latex rubber gloves^21,22,33,34^. This suggests that ZDEC is a major factor in inducing hypersensitivity from latex rubber gloves^21^. The hypersensitivity to ZDEC is further affirmed by reports showing that 0.5% and 1.0% ZDEC coated polyurethane discs implanted into rat abdominal wall caused a significantly thicker foreign body capsule than nontoxic, non-coated polyurethane discs^35^. However, these studies only described the cytotoxicity of ZDEC indirectly, without fully understanding what ZDEC was doing to the cells at a molecular level^21,36^. From this study, ZDEC (concentration greater than 0.0001%) caused cell damage, resulting in low metabolic activity, apoptosis and cell death of HLEC.

Damaged cells can release various cytokines, which recruit immune cells to the site, ultimately leading to inflammation^4,37^. Cytokine release from the cells varied, depending on the toxin evaluated and the toxin concentration. For cells exposed to ZDEC the release of the cytokines IL-1β, IL-8, and TNF-α decreased as the concentration of ZDEC increased. This could have been the result of cell toxicity as a decrease in cell metabolic activity would have reduced the production in the cell of these cytokines. For IL-6 a similar mechanism of cytokine inhibition could have caused the sharp drop in the amount of cytokine released at the highest concentration tested. However, at 0.001% ZDEC may have stimulated IL-6 cytokine production. Increasing the concentration of BAK on the other hand caused an increased release of the cytokines IL-1β, IL-8, and TNF-α for one or more dose concentrations, but did not affect the release of IL-6. Depending on the toxin evaluated, increased toxicity caused either an increase or a decrease in the release of inflammatory cytokines.

The release of mostly live cells from a material, as opposed to mostly apoptotic cells, could represent varying issues with respect to a material’s biocompatibility. Live cells could reattach to other locations on the biomaterial. In the case of an intraocular lens implant, if the cells move centrally towards the visual axis it could contribute to the formation of PCO. Contrarily, if there is extensive cellular death, then inflammation may ensue. In addition, it has been reported that apoptosis of LEC post-cataract surgery can be beneficial as it would prevent cell growth and movement of LEC to the central visual axis^11,38^. Thus, it seems there needs to be an optimal level of apoptosis for maximal long-term tolerability of an intraocular lens implant. If the implant material is non-toxic, HLEC can attach to the implant and can grow, migrate and contribute to PCO. On the other hand, if the material is too toxic too much cell damage could occur causing excessive intraocular inflammation.

Another cell line, THP-1 derived macrophage cells, has been studied for cytotoxicity after adhesion to silicone films^39^. Macrophages were grown in 10% serum vs the 20% serum in the HLEC of this investigation. Similar to the response of HLEC in this study, dose response toxicity to ZDEC and BAK occurred and substantial toxicity to the cells were shown at 0.01% ZDEC and 0.1% BAK concentration. Comparing the sensitivity of various cell lines to each other could be undertaken in future investigations by performing the toxicity testing at similar serum levels as serum proteins can bind to toxins and other bioactive substances^40,41^. Both HLEC and macrophage adhesion to ocular implants are important as HLEC adhesion causes posterior capsular opacification and macrophage adhesion results in a foreign-body reaction against the ocular implant^42^.

For cytotoxicity testing, it is necessary for a method or a battery of tests to be developed which can assess the viability of cells adhered to a surface, to broaden our understanding of the cellular interactions to a material. Ultimately, this will contribute to the creation of more novel and compatible biomaterials. Both the assessment of the fluorescent dyes using confocal microscopy and the evaluation of resazurin reduction were able to detect the cytotoxicity for ZDEC and BAK. Cytokine release decreased or increased depending on the toxin and the toxin concentration.

Future evaluations of new materials for IOL can utilize the methods used in this investigation to screen out chemicals and leachables from IOL surfaces that are too toxic to allow for IOL biocompatibility. In addition, some chemicals may be identified that may cause the release of HLEC over time without causing toxicity to the cells. It is these types of materials that can be identified in the future that may prevent PCO.

## Materials and methods

### Cell culture

HLEC (CRL-11421, American Type Culture Collection, Manassas, VA, USA) were grown in minimum essential medium (MEM) with l-glutamine and Earle’s salts (Gibco, Invitrogen, CA, USA) supplemented with 100 IU/mL of Penicillin, 100 μg/mL of Streptomycin (Invitrogen) and 20% fetal bovine serum (Invitrogen). Cells were incubated at 37 °C with 5% CO_2_ and grown to confluence with media changes every 2–3 days. At confluence, the cells were dissociated with TrypLE™ Express (Invitrogen) to be seeded onto treated silicone discs.

### Test surface preparation

Silicone discs were cut from medical grade silicone sheeting (Specialty Manufacturing Inc., Saginaw, MI, USA). Prior to use, the discs were soaked in ethanol (70%) for at least 1 h for sterilization followed by a soak in phosphate buffered saline (PBS, Lonza, Walkersville, MD USA). A silicone disc was placed into each well of a 24-well tissue culture plate (BD Biosciences, Mississauga, ON, Canada). Varying percent concentrations of test substances in PBS (1 mL per well) were added to the wells. The test substances were ZDEC (in DMSO and added at 100-fold volume dilution to PBS in each well over the test sample), BAK (Sigma-Aldrich, Oakville, ON, Canada). PBS was used as a control. The wells were incubated for 24 h to allow adsorption of the test substance onto the silicone. After the 24 h soak the solutions were removed and replaced with 1 mL MEM with serum.

### Cell exposure to material surfaces

Monolayer cultures on control silicone surfaces were seeded onto discs exposed to PBS but treated in the same manner as the test silicone surfaces with regards to handling and medium change. Each control and treated silicone disc was seeded with 5 × 10^5^ HLEC in 1 mL MEM with serum. The cells were incubated at 37 °C with 5% CO_2_ for 18 h prior to evaluation for cell viability using confocal microscopy, inflammatory cytokine release and metabolic activity.

### Confocal analysis of cell viability with three fluorescent dyes

After the 18 h incubation of HLEC, the ZDEC and BAK treated silicone discs were removed from the wells. The discs were stained with annexin binding buffer solution of ethidium homodimer (4 µM), calcein AM (2 µM), and annexin V (10 µL in 500 µL buffer)– Alexa Fluor 647 conjugate (Invitrogen) at room temperature in Petri dishes with glass bottom cover slips (MatTek Corporations, Ashland, MA, USA). Cells were then transferred onto another MatTek dish containing 500 µL of staining solution for imaging with the confocal laser microscope. The non-adhered HLEC from the staining wash and the adhered cells from the sample could then be evaluated. The fluorescence of ethidium, calcein and annexin V was then examined with an Axiovert 100 microscope with a 40× water-immersion C-Apochromat objective with a confocal laser scanning 510 system (Carl Zeiss Inc., Germany). The wavelengths (excitation/emission) for ethidium homodimer-1, calcein and annexin V were, 543/617 nm, 488/517 nm, 633/665 nm, respectively.

Each silicone disc was divided into five sectors and three random microscopic fields in each sector was selected and counted (total of 15 fields in each experiment). A count of live, apoptotic cells and dead in each field was performed. Test samples were evaluated from two independent experiments.

### Release of inflammatory cytokines

After the 18-h incubation of the cells on the ZDEC and BAK treated silicone discs, the media was pipetted into sterile polypropylene tubes, at – 80 °C until evaluated for cytokine analysis. Cytokines were quantified using the MesoScale Discovery^®^ (MSD^®^) QuickPlex SQ 120 (Rockville, MD, USA). The MSD^®^ Human Proinflammatory Panel II (4-Plex) V-Plex assay kit quantified four proinflammatory cytokines: TNF-α, IL-1β, IL-6, and IL-8. Each MSD kit evaluated has minimal sensitivity of IL-1β, = 0.17, IL-8 = 0.14, IL-6 = 0.09, TNF-α = 0.13 pg/mL. The experiments were performed in triplicate in two separate experiments.

### alamarBlue assay

After incubation of the HLEC on the control and treated silicone surfaces for 18 h, the media was removed. 1 mL of 10% alamarBlue prepared in MEM without serum was added to each well and incubated for 4 h at 37 °C with 5% CO_2_. A SpectraMax fluorescence multi-well plate reader (Molecular Devices, Sunnyvale, CA, USA) was used to determine the fluorescence of each well at 530/590 nm excitation/emission. The experiments were performed in triplicate in two separate experiments.

### Statistics

Statistical significance was performed using GraphPad Prism 9 statistical software (San Diego, Ca, USA). Normality of the data was evaluated, and equality of variances were assessed. Statistical differences between means were performed by the Kruskal–Wallis test with Dunn’s multiple comparisons test for the cell counts of live, dead and apoptotic cells. For assessment of the differences in cytokine release from cells exposed to each toxin either a standard ANOVA or a Welch ANOVA was performed as determined by the results of the equality of variances test and comparisons between means were evaluated by the Dunnett’s multiple comparison test.

Differences were considered significant when the probability was less than 0.05.

## Supplementary Information

Supplementary Information 1.

Supplementary Information 2.

## Data Availability

Data available upon reasonable request.
