# Supplementary figures and images for: In vitro assessment of the biocompatibility of chemically treated silicone materials with human lens epithelial cells
Source: Sci Rep. 2022 Mar 17;12:4649. doi: 10.1038/s41598-022-08443-2 (PMC8931081; doi:10.1038/s41598-022-08443-2)

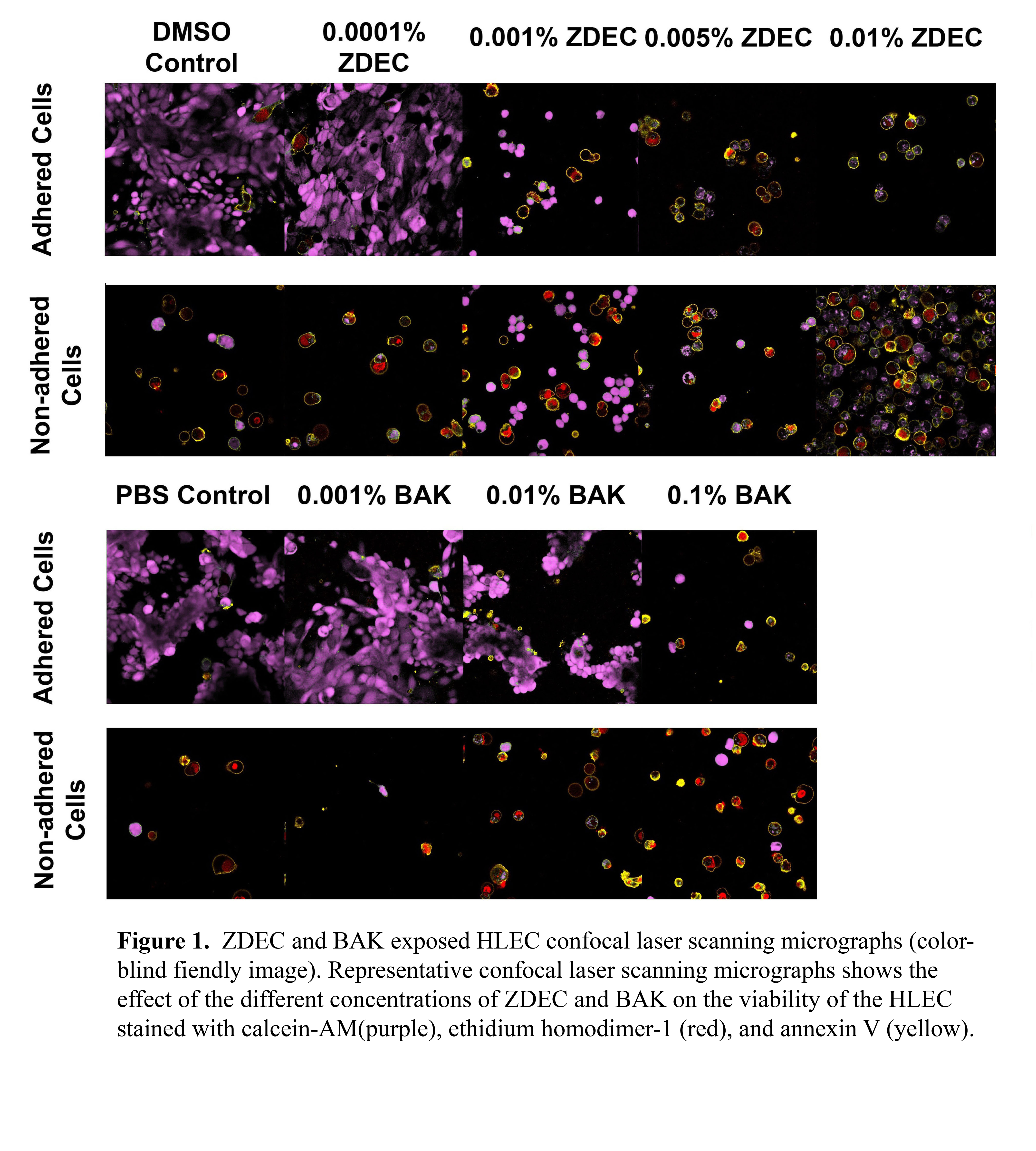

Supplement: Supplementary file 1 — Supplementary Information 1. [file 41598_2022_8443_MOESM1_ESM.tif]

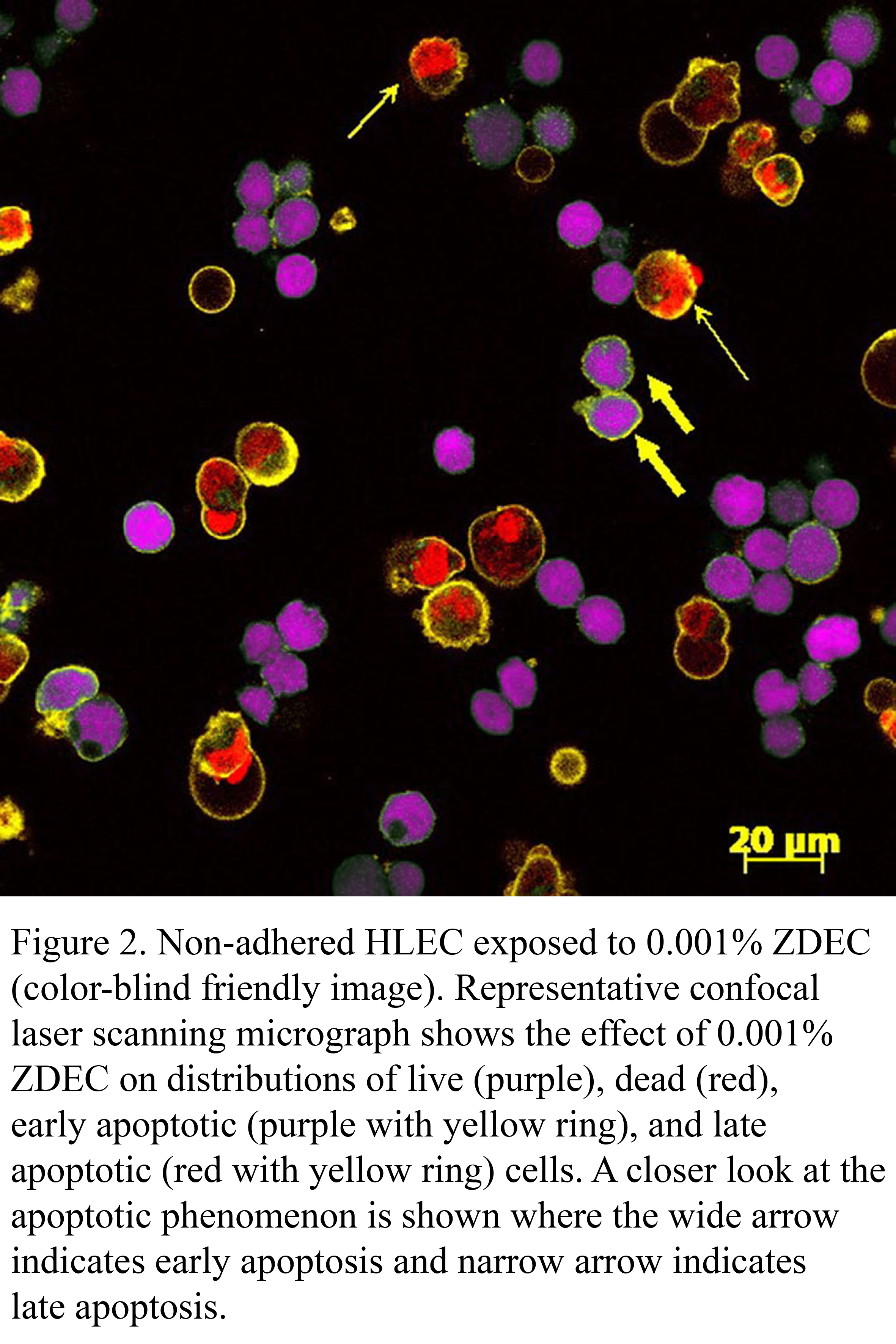

Supplement: Supplementary file 2 — Supplementary Information 2. [file 41598_2022_8443_MOESM2_ESM.tif]
